# Collective effect of self-learning and social learning on language dynamics: a naming game approach in social networks

**DOI:** 10.1098/rsif.2024.0406

**Published:** 2024-12-04

**Authors:** Tao Wen, Yu-wang Chen, Renaud Lambiotte

**Affiliations:** ^1^ Decision and Cognitive Sciences Research Centre,The University of Manchester, Manchester M15 6PB, UK; ^2^ Alan Turing Institute, London, NW1 2DB, UK; ^3^ Mathematical Institute, University of Oxford, Oxford, OX2 6GG, UK

**Keywords:** naming game, generalized Bayesian inference, social network, social behaviour, language dynamics

## Abstract

Linguistic rules form the cornerstone of human communication, enabling people to understand and interact with one another effectively. However, there are always irregular exceptions to regular rules, with one of the most notable being the past tense of verbs in English. In this work, a naming game approach is developed to investigate the collective effect of social behaviours on language dynamics, which encompasses social learning, self-learning with preference and forgetting due to memory constraints. Two features that pertain to individuals’ influential ability and affinity are introduced to assess an individual’s role of social influence and discount the information they communicate in the Bayesian inference-based social learning model. Our findings suggest that network heterogeneity and community structure significantly impact language dynamics, as evidenced in synthetic and real-world networks. Furthermore, self-learning significantly enhances the process of language regularization, while forgetting has a relatively minor impact. The results highlight the substantial influence of network structure and social behaviours on the transition of opinions, from consensus to polarization, demonstrating its importance in language dynamics. This work sheds new light on how individual learners adopt language rules through the lenses of complexity science and decision science, advancing our understanding of language dynamics.

## 1. Introduction

There are a multitude of human languages in the world, all capable of expressing a wide range of concepts, from the concrete to the abstract. The key factor that enables mutual understanding between communicating parties is the fundamental linguistic rules shared by human languages [1], which encompass both regular and irregular elements. Importantly, irregular exceptions to linguistic rules can be acquired and accepted without causing significant ambiguity in the conversation [2], as demonstrated in the past tense of verbs in English [3,4]. Specifically, regularity typically refers to the application of the modern *-ed* rule, such as help/helped, while other forms are considered irregular, like go/went. Irregular verbs, though constituting less than 3% of all verbs in modern English, remain prevalent in daily communication. Notably, the top 10 most frequently used verbs in English are irregular [5]. The presence of irregular verbs has been found to be associated with their frequency of use and class membership [5–8]. In addition, these linguistic rules have evolved in intricate social contexts, portraying language as a dynamic system, commonly referred to as language dynamics. To investigate language dynamics, agent-based models [9–13] have proven to be instrumental, especially within the framework of the naming game [14–16]. In the naming game, individuals engage in pairwise interactions and negotiate conventional forms corresponding to a predefined set of meanings over time. In each pairwise interaction, two individuals are randomly selected from the population and assigned the roles of speaker and hearer to interact about a specific meaning. In instances where initial communication is unsuccessful, the population can gradually converge on shared forms to reference specific meanings [17,18].

In the context of language dynamics, initially, individuals could adopt only one of two states: regular rules or irregular exceptions [19]. However, as people can use both regular and irregular verb forms over a period of time, this model has been expanded to encompass three states, introducing a new mixed state to describe this scenario [19,20]. This extended framework has revealed that the distinction between speaker and hearer does not have a discernible effect on collective behaviour. In addition, a discontinuous frequency transition has been observed in the three-state model [19], which is absent in the two-state model. Moreover, biased child learners were introduced to characterize preferences for regularity during early language acquisition [21], and various forms of memory constraints have been incorporated to illustrate their role in children’s over-regularization errors. When these factors are combined with the frequency of lemma use, the regularities observed in natural language, that is, verbs used frequently tend to maintain their irregular forms, have been observed.

However, it is crucial to recognize that individuals exist within stable social structures and dynamics, as opposed to isolation [22]. Hence, individuals are more likely to engage in the exchange of opinions on linguistic rules with their connected neighbours, such as close friends and family members, rather than randomly selecting individuals from the entire society [19,20]. In addition, the uncertainty associated with an individual’s decision-making [23] can significantly influence the linguistic rules they choose to adopt. Even when individuals possess complete knowledge of a verb, they may hold varying beliefs about each state to express the level of support, rather than simply adopting or rejecting a particular state. In other words, individuals will not categorically fall into either the regular state, irregular state, or mixed state, as seen in prior naming game models [19,20]. Instead, they will express their support for diverse linguistic rules through beliefs, a concept commonly used in classical opinion dynamics models [24–27], such as the DeGroot model and the Hegselmann–Krause model. Moreover, when individuals receive information from others in the interaction, they accept discounted beliefs based on the social influence and expertise of others [28,29]. This concept of trust between individuals [30,31] has been ignored by previous models of language dynamics. Furthermore, in real-world scenarios where numerous verbs exist, it is improbable for an individual to possess equivalent knowledge of all verbs due to gaps in their knowledge base [32]. For example, individuals may tend to use both the regular form learn/learned and the irregular form dream/dreamt, even though both verbs have two spellings of the past tense. Therefore, language dynamics in multi-word cases, a common scenario in language use [18,33], need to be taken into account.

Therefore, in this work, we introduce a naming game approach to investigate the impact of social behaviours and network structures on language dynamics within a society. Specifically, individuals and their relationships are represented using nodes and edges in a social network, meaning that activities and information exchanges are restricted to those who are directly connected in the social network [27]. By incorporating a generalized Bayesian inference model [34,35], each individual can possess a personal belief system to express the level of support or preference for any form of verbs in multi-word cases, eliminating the need for a third mixed state to describe the simultaneous support for both regular and irregular forms of a verb. This belief evolves over time through the social learning process. Additionally, our model considers individual self-learning with preferences, similar to how child learners exhibit a bias toward regular verbs [20], as well as the role of memory constraints for forgetting [36,37]. These factors are crucial for comprehensively modelling the process of belief updates regarding linguistic rules. Given that individuals do not possess complete knowledge [38], the information they provide is naturally constrained by their position in the network and their existing knowledge, characterized by their external and internal attributes. The external attribute, quantified by weight, signifies an individual’s importance and influential ability within the network under generic scenarios [39,40]. The internal attribute, denoted by reliability, relates to the affinity between individuals because like-minded individuals whose beliefs align with their own perspectives are typically considered more trustworthy. These attributes further affect the sequence and direction in which individuals learn from their neighbours in the network.

Various synthetic network structures are employed to investigate the collective effect of social behaviours in the naming game model, including scale-free networks, random networks and small-world networks. A family of networks [41], sharing the same average connectivity while featuring tunable degrees of heterogeneity, are examined to show how network heterogeneity fosters the consensus process among individuals. Furthermore, real-world social networks and Lancichinetti–Fortunato–Radicchi (LFR) benchmark networks with known community structures [42] are employed to highlight the influence of community structure on language dynamics. Several indicators are also established to describe the process of convergence, consensus and regularization of individuals in networks [43]. Furthermore, the impact of self-learning and forgetting behaviours in different synthetic networks is examined, shedding light on their implications. The results demonstrate that the topological structure of networks significantly influences group opinions (polarization or consensus), which can be attributed to network heterogeneity or the presence of community structures. Moreover, self-learning has the potential to significantly enhance the language regularization process, whereas forgetting, mainly attributed to memory constraints, has a relatively minor impact. However, both behaviours contribute to the promotion of consensus-reaching. This insight can be valuable for group leaders and policymakers seeking to cultivate consensus and enhance effective communication, emphasizing the intricate interplay between network structure and language dynamics that affects several aspects of societal communication.

## 2. Methods

**Figure 1.**
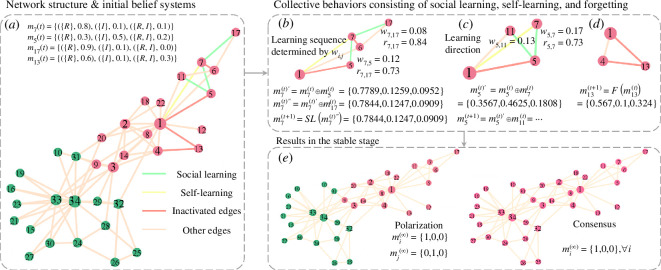
The framework of the naming game model within the social network structure, where Zachary’s karate club network is used as an example. (*a*) The topological structure of the network and belief systems of four individuals whose interaction involves only $n=n_{0}=1$ verb are given, where the colour of nodes indicates their community, while the colour of edges indicates activities, with green, yellow and red denoting social learning, self-learning and inactivated edge, respectively. (*b*) The ego network of individual 7 is selected to illustrate social learning with a learning sequence determined by the weight $w_{ij}$ and self-learning (indicated by SL(⋅)) with α=5%. (*c*) The ego network of individual 5 is selected to highlight the effect of learning direction, where $m_{5}^{\left(t\right)}⊕︎m_{7}^{\left(t\right)}\neq m_{7}^{\left(t\right)}⊕︎m_{5}^{\left(t\right)}$. (*d*) The ego network of individual 13 is chosen to demonstrate the forgetting (indicated by F(⋅)) with β=4%, as this individual does not engage in any learning process. (*e*) Two typical results, polarization and consensus, are showcased, where the colour of nodes indicates the converged belief of individuals.

Within a given network denoted as G(N,E), there is a set of individuals N={1,⋯,|N|} connected by set of social interactions E={(i,j):i,j∈N}. At time t, each edge (i,j) is activated with probability f, allowing two connected individuals to engage in an interaction and update their opinions. In this work, the roles of speaker and hearer are assigned to the two connected individuals with equal probability, adhering to a neutral strategy [18], because of their indistinguishable effect on collective behaviour [19]. If the edge is activated, two connected individuals could either (i) engage in information exchange to update their beliefs by social learning with probability $p_{s}$ or (ii) undertake self-learning with probability $1-p_{s}$ to reinforce one state [37] similar to child learners biased towards regular verbs [20]. Otherwise, if an individual is not actively engaged in learning (less than η for both types of learning), he or she may experience memory limitations with probability $p_{f}$, resulting in the forgetting of existing knowledge. The detailed process consisting of social learning, self-learning and forgetting in social networks is described in figure 1.

For each individual i, the belief $m_{i}^{\left(t\right)}$ held at time t is characterized by the belief function in Dempster–Shafer theory. Dempster–Shafer theory [44,45] is a generalized Bayesian inference model that assigns belief functions to sets of events to express the uncertainty supporting an arbitrary event. More details about the basic concepts of Dempster–Shafer theory can be found in the appendix. In the context of language dynamics, we consider a scenario with n verbs, each of which has both regular R and irregular I forms. Specifically, the frame of discernment for the linguistic rule adopted by each individual can be expressed as


(2.1) $$\Theta =\{R_{1},R_{2},\cdots ,R_{n},I_{1},I_{2},\cdots ,I_{n}\},$$

which is the set of all possible verb forms that an individual can adopt, each indicating the adoption of a form of a specific verb. Mathematically, it is a finite collection encompassing mutually exclusive and collectively exhaustive elements due to their empty intersection. Its power set $2^{\Theta }$ or P(Θ) represents all possible combinations of elements, that is, all possible combinations of verb forms an individual can adopt, and it is defined as


(2.2) $$\begin{array}{rl} P(\Theta )=2^{\Theta }= & \{\{R_{1}\},\{\cdots \},\{R_{n}\},\{I_{1}\},\{\cdots \},\{I_{n}\},\\ & \{R_{1},R_{2}\},\{R_{1},I_{1}\},\{\cdots \},\{R_{n},I_{n}\},\\ & \{R_{1},R_{2},I_{1}\},\{\cdots \},\{R_{n},I_{n-1},I_{n}\},\\ & \{\cdots \},\\ & \{R_{1},\cdots ,R_{n},I_{1},\cdots ,I_{n}\}=\Theta ,\\ & \emptyset \}. \end{array}$$

In addition, it contains all subsets of the frame of discernment Θ with $2^{2n}$ propositions, including the empty set ∅ and Θ itself. The degree of support for each proposition $m:2^{\Theta }\to [0,1]$ is described by the belief function [44], satisfying


(2.3) $$m(\emptyset )=0,\sum_{A\subseteq \Theta }m(A)=1,$$

which is assigned to the subsets of elements in the power set rather than individual elements. Here, m(A) measures the degree of belief exactly assigned to the proposition $A\in 2^{\Theta }$ and no smaller subset, indicating how strongly the individual supports the proposition. The subsets $A\in 2^{\Theta }$ that satisfy m(A)>0 are called focal elements. For example, full support to a singleton proposition $m(R_{1})=1$ indicates that the individual currently has made a commitment to using the regular form of the first verb and does not have preferences for any forms of other verbs, regardless of whether this individual knows other verbs. In the belief system, simultaneous preferences for both regular and irregular forms can be expressed in terms of belief functions, such as $m(R_{1},I_{1})$, rather than Bayesian probability functions, thereby avoiding the introduction of a third mixed state, as seen in the previous models [19,20]. Specifically, $m(R_{1},I_{1})=1$ indicates complete simultaneous preferences for both regular and irregular forms, and it cannot be assigned to any subsets, meaning it cannot specify a preference for the regular $m(R_{1})$ or irregular $m(I_{1})$ forms of the first verb at the current stage. In this case, each individual can perceive the degree of support for different forms of multiple verbs from others. The social network and four individuals’ belief functions with one verb are exemplified in figure 1*a*.

During each social learning on activated edges, two connected individuals may mention only a limited number of verbs, resulting in varying $n_{0}\leq n$ in the interaction. Therefore, the frame of discernment, composed of any number of verbs, can be adopted in social learning for this edge. For example, if only $n_{0}=1$ verb is mentioned in the interaction on edge (i,j), the frame of discernment is denoted as $\Theta_{ij}=\{R_{1},I_{1}\}$. In the scenario where individual i perceives the degree of support for both $R_{1}$ and $I_{1}$ from individual j, the corresponding belief function for this proposition should satisfy $m_{j}\left(\{R_{1},I_{1}\}\right)\neq 0$. The belief function will reduce to the probability function if only the belief function assigned to the singleton proposition is larger than 0. In contrast to Bayesian theory, this approach [44] not only represents preferences for linguistic rules but also explicitly represents uncertainty and ignorance, which is particularly useful for dealing with situations where individuals hold conflicting or incomplete information.

### 2.1. Generalized Bayesian inference-based social learning

On the already-activated edge, two connected individuals have the opportunity to exchange and assess information from each other with probability $p_{s}$, leading to the update of their personal opinions through social learning. However, due to inherent knowledge limitations [38], each individual evaluates the information delivered by others based on their external and internal attributes, thereby learning based on discounted information. In this work, information is discounted by two attributes: weight and reliability. The first attribute involves the extrinsic feature of an individual, specifically the relative importance of the individual in society as perceived by another individual [46]. This relative importance of individual j assigned by individual i is quantified by the weight $w_{ij}$, which restricts the belief held by individual j in the process of information exchange. Therefore, each individual operates within defined bounds dictated by its weight concerning another individual. In essence, it embodies the preconceived perception or assumptions that individuals may hold about each other before any notable events occur. In the context of social networks, this concept can be reflected in the network structure and quantified by centrality [39,40]. In this work, it is assumed that each individual can only perceive their local information, thus, the weight is determined by the degree centrality,


(2.4) $$\begin{array}{ccc} & w_{ij}=d_{j}/\sum_{j\in \Gamma (i)}d_{j}, & \end{array}$$

where Γ(i) denotes the neighbourhood set of individual i. Notably, $w_{ij}$ may not be equivalent to $w_{ji}$ due to the different sets of acquaintances (neighbours).

**Figure 2.**
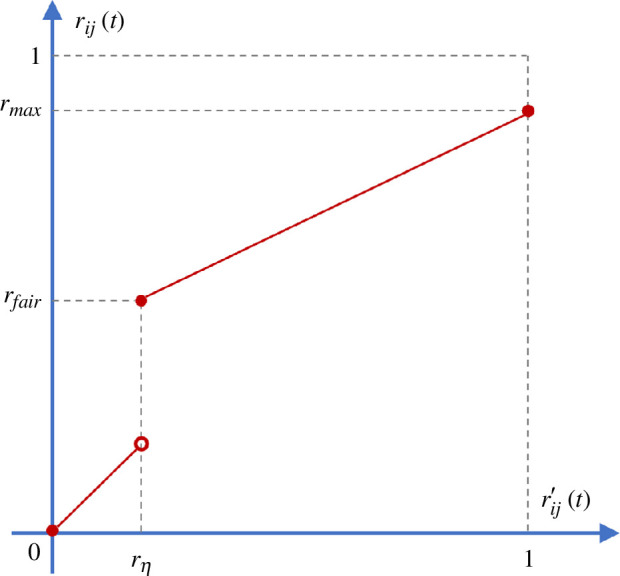
The mapping mechanism to transform original reliability $r^{\prime }$ to a meaningful scale r.


The second factor influencing information discounting is the intrinsic feature, which gauges the affinity of others [47]. In detail, the affinity of individual j to individual i is measured by their shared beliefs, indicated by the reliability $r_{ij}$. The cosine similarity is adopted in this work to measure the similarity of the beliefs following a particular event,


(2.5) $$\begin{array}{ccc} & r_{ij}^{\prime }(t)=s\left(m_{i}^{\left(t\right)},m_{j}^{\left(t\right)}\right), & \end{array}$$

which calculates the normalized dot product between two belief functions. Reliability is not a static attribute but evolves over time as belief functions change. In social networks, individuals who are connected often share a certain level of friendship. Research has shown that key behaviours contributing to the formation and maintenance of friendships encompass qualities such as supportiveness, positivity and interaction [48]. Therefore, a fundamental basis for friendship lies in the fair assessment of reliability, which should not fall below a predefined lower threshold. Furthermore, it is common for individuals to assign a finite upper limit to the reliability of their friends rather than assigning the maximal value $r_{ij}=1$. This practice aligns with the understanding that no single individual possesses exhaustive knowledge across all subjects [38]. Therefore, the mapping mechanism, as depicted in figure 2, is mathematically defined as follows:

(2.6) $$\begin{array}{r} r_{ij}(t)=\left\{ \begin{array}{cc} r_{\text{fair}}+\frac{\left(r_{\text{max}}-r_{\text{fair}}\right)\left(r_{ij}^{\prime }(t)-r_{\eta }\right)}{1-r_{\eta }}, & \text{if }r_{ij}^{\prime }(t)\geq r_{\eta }\\ r_{ij}^{\prime }(t), & \text{if }r_{ij}^{\prime }(t)<r_{\eta } \end{array}\right., \end{array}$$

where the fair reliability $r_{\text{fair}}=0.5$ implies that the belief is considered to be half correct and half incorrect, $r_{\eta }$ indicates the threshold needed to achieve fair reliability, and $r_{\text{max}}$ indicates the upper limit to the reliability.

In pairwise interaction between individuals, if two individuals have highly conflicting or opposing beliefs, they typically assign low reliability to one another because they prefer to trust their own beliefs. However, one of these opposing beliefs may be more widely recognized by the public and more suitable for the current scenario when additional information is introduced from the public. Therefore, if there is an opportunity to contact a third individual to perceive additional information, an individual can assess whether their own belief aligns with the public’s perception, thereby adjusting their confidence and modifying the reliability they assign to others. Specifically, when individuals i and $j_{1}$ with conflicting beliefs engage in social learning, they assign low reliability to each other due to their conflicting views. However, when individual i encounters a third person, $j_{2}$, who also holds the opposing belief, individual i may recognize that this belief held by the group consisting of $j_{1}$ and $j_{2}$ is more aligned with the public’s perception and more suitable for the current scenario. In this case, individual i will assign $j_{2}$ high reliability, despite their conflicting beliefs, allowing individual i to adjust their own beliefs accordingly to avoid being overconfident in themselves and deviating from the public belief. When individual i meets the next individual, this process continues until this round of social learning with others is completed. This rationale motivates us to introduce an in-group recursive approach to determining reliability, which helps prevent individuals from insisting on unsuitable beliefs when confronted with opposing views from others,


(2.7) $$\begin{array}{r} r_{ij_{k}}^{\prime }(t)=\left\{ \begin{array}{cc} s\left(m_{i}^{\left(t\right)},m_{j_{k}}^{\left(t\right)}\right), & \text{if }k=1\\ \frac{s\left(m_{i}^{\left(t\right)},m_{j_{k}}^{\left(t\right)}\right)}{2}+\frac{\sum_{j_{a},j_{b}\in \Gamma (i{)}_{k},a<b}s\left(m_{j_{a}}^{\left(t\right)},m_{j_{b}}^{\left(t\right)}\right)}{k\times (k-1)}, & \text{if }k\neq 1 \end{array}\right., \end{array}$$

where $\Gamma (i{)}_{k}=\{j_{1},j_{2}\cdots \;,j_{k}\}$ consists of the first k neighbours with whom individual i exchanges information. The difference between weight and reliability resembles the distinction between the ‘nature’ and ‘nurture’ factors influencing individuals, a significance demonstrated in a recent study [49].

After determining the weight $w_{ij}$ and reliability $r_{ij}$ of each individual, the weighted evidential reasoning approach with reliability [50], a nonlinear combination method, is employed to model individual social learning process in network scenarios,


(2.8) $$m_{i}^{\left(t+1\right)}=m_{i}^{\left(t\right)}⊕m_{j}^{\left(t\right)}⊕\cdots ,$$

where $m_{i}^{\left(t\right)}$ represents the belief function of individual i at time t. An example of the social learning process is illustrated in figure 1*b*. Specifically, within the context of social learning from ego individual i in the network, the weighted belief degree with reliability of individual j ($\in \{i,\Gamma_{i}\}$) is defined as


(2.9) $$\begin{array}{r} {\hat{m}}_{j}(\theta )=\left\{ \begin{array}{cc} 0, & \theta =\emptyset \\ c_{ij}m_{i}(\theta ), & \theta \subseteq 2^{\Theta },\theta \neq \emptyset \\ 1-c_{ij}, & \theta =P(\Theta ) \end{array}\right., \end{array}$$

where $c_{ij}=w_{ij}/\left(1+w_{ij}-r_{ij}\right)$ is the combined discounting coefficient, and ${\hat{m}}_{j}(P(\Theta ))$ is the remaining support left uncommitted by individual j due to weight and reliability. In this context, $w_{ij}$ and $r_{ij},\forall j$ are not necessarily normalized [50]. Additionally, we assume $w_{ii}=r_{ii}=1$ to reflect the inherent trust an individual places in their own information and beliefs. The weighted belief degree with reliability combined by the first l−1 individuals and individual l, i.e. ${\hat{m}}_{i}^{e(l)}={\hat{m}}_{i}^{e(l-1)}⊕︎{\hat{m}}_{l}$, is defined by


(2.10) $$\begin{array}{rl} {\tilde{m}}_{e(l)}(\theta )= & \left[{\hat{m}}_{l}(P(\Theta )){\hat{m}}_{e(l-1)}(\theta )+{\hat{m}}_{e(l-1)}(P(\Theta )){\hat{m}}_{l}(\theta )\right]\\ & +\sum_{B⋂C=\theta }{\hat{m}}_{e(l-1)}(B){\hat{m}}_{l}(C),\\ {\tilde{m}}_{e(l)}(P(\Theta ))= & {\hat{m}}_{l}(P(\Theta )){\hat{m}}_{e(l-1)}(P(\Theta )), \end{array}$$

where e(l) indicates the first l individuals, ${\hat{m}}_{e(1)}(\theta )={\hat{m}}_{1}(\theta )$ and ${\hat{m}}_{e(1)}(P(\Theta ))={\hat{m}}_{1}(P(\Theta ))$. In this case, ${\hat{m}}_{e(l)}(\theta )$ is the combined belief function of the first l individuals. The weighted belief degree with reliability is renormalized by


(2.11) $$\begin{array}{rl} {\hat{m}}_{e(l)}(\theta ) & =\frac{{\tilde{m}}_{e(l)}(\theta )}{\sum_{\theta \subseteq 2^{\Theta }}{\tilde{m}}_{e(l)}(\theta )+{\tilde{m}}_{e(l)}(P(\Theta ))},\\ {\hat{m}}_{e(l)}(P(\Theta )) & =\frac{{\tilde{m}}_{e(l)}(P(\Theta ))}{\sum_{\theta \subseteq 2^{\Theta }}{\tilde{m}}_{e(l)}(\theta )+{\tilde{m}}_{e(l)}(P(\Theta ))}, \end{array}$$

in each iteration. After exchanging information and learning from its l−1 neighbours, the belief function can be obtained by


(2.12) $$m_{e(l)}(\theta )=\frac{{\hat{m}}_{e(l)}(\theta )}{1-{\hat{m}}_{e(l)}(P(\Theta ))},\theta \subseteq 2^{\Theta }.$$

It is important to note that the sequence in which individuals exchange information and learn from neighbours will affect the results of belief updating, that is, $\left(m_{i}^{\left(t\right)}⊕︎m_{j}^{\left(t\right)}\right)⊕︎m_{k}^{\left(t\right)}\neq \left(m_{i}^{\left(t\right)}⊕︎m_{k}^{\left(t\right)}\right)⊕︎m_{j}^{\left(t\right)}$. The sequence of social learning is determined by the importance of neighbours, quantified by the weight $w_{ij}$ in this work. This aligns with reality, where individuals typically encounter information first from sources that hold greater personal significance to them. Therefore, $\Gamma (i{)}_{k}$ in (2.7) includes the top k most important neighbours of individual i. In addition to the sequence of learning, the direction of learning is also considered, with updated beliefs differing depending on the direction of learning between two individuals $m_{i}^{\left(t\right)}⊕︎m_{j}^{\left(t\right)}\neq m_{j}^{\left(t\right)}⊕︎m_{i}^{\left(t\right)}$, as shown by the different belief functions after the interaction between individuals 5 and 7 in figure 1*b*,*c*. This generalized Bayesian inference model takes into account both extrinsic and intrinsic features of individuals and incorporates learning direction and sequence to better reflect real-world scenarios [51,52].

Overall, social learning follows an asynchronous update approach [52] after determining the weight and reliability. This means that $m_{i}^{\left(t\right)}$ is used to combine other belief functions at time t, even if it has already been updated to $m_{i}^{\left(t,n\right)}$. Given this asynchronous nature, the sequence in which ego individuals are chosen does not affect the social learning results.

### 2.2. Self-learning with preference

A preference for learning regular verbs has been found in the learning process, particularly observed in child learners during their early stages of language acquisition [21]. This phenomenon has been examined in naming game-based models [19,20]. In this work, individuals on the activated interaction reinforce their beliefs [37] about regular forms through self-learning with probability $1-p_{s}$. When dealing with multiple verbs, a verb is randomly chosen, which means that R and I can be any element in $\left\{R_{1},R_{2},\cdots \;,R_{n}\right\}$ and $\left\{I_{1},I_{2},\cdots \;,I_{n}\right\}$, respectively. This process, SL(⋅) shown in figure 1*b*, can be characterized by


(2.13) $$\begin{array}{rl} m_{i}^{\left(t+1\right)}(R) & =m_{i}^{\left(t\right)}(R)+\alpha m_{i}^{\left(t\right)}(I),\\ m_{i}^{\left(t+1\right)}(I) & =\left(1-\alpha \right)m_{i}^{\left(t\right)}(I), \end{array}$$

where α represents the self-learning rate of individuals. However, if $m_{i}^{\left(t\right)}(I)=1$, which means that individual i has already made a commitment to using one irregular form, this individual no longer engages in self-learning to reinforce R. In cases where an individual has the opportunity to learn from neighbours and engage in self-learning through different activated interactions, the individual first acquires information from his/her neighbours before proceeding with self-learning.

### 2.3. Forgetting due to memory constraints

Individuals within the activated connection possess the capacity to update their beliefs. However, many individuals do not actively engage in the learning process (including social learning and self-learning) in the network due to the limited number of activated edges. These individuals will experience memory limitations that can result in the forgetting of current beliefs [36,37]. In this work, individuals who have not yet reached stability $\max_{\theta }\;m_{i}^{\left(t+1\right)}(\theta )\neq 1$ and have participated in learning (whether through social learning or self-learning) fewer than η in the current round are susceptible to forgetting their current beliefs. Therefore, individual i will forget the proposition in which he or she currently holds the strongest belief and shift towards m(Θ) with probability $p_{f}$, which is described by


(2.14) $$\begin{array}{rl} m_{i}^{\left(t+1\right)}(\Theta ) & =m_{i}^{\left(t\right)}(\Theta )+\beta m_{i}^{\left(t\right)}(b_{f}),\\ m_{i}^{\left(t+1\right)}(b_{f}) & =\left(1-\beta \right)m_{i}^{\left(t\right)}(b_{f}), \end{array}$$

where $b_{f}={arg\;max}_{\chi \in 2^{\Theta }\setminus \Theta }m_{i}^{\left(t\right)}(\chi )$. This process is demonstrated by F(⋅) in figure 1*d*. Any required forgetting due to memory constraints will occur after individuals have engaged in social learning and self-learning, as the number of times it has been engaged in learning needs to be measured. After this, the belief updating process at time t is concluded.

## 3. Results

A scenario with two verbs ($n=n_{0}=2$) with regular R and irregular I forms involved in the interaction is incorporated to illustrate the process developed in this work. In this case, the frame of discernment is $\Theta =\left\{R_{1},R_{2},I_{1},I_{2}\right\}$, and its power set is represented as


$$\begin{array}{rl} 2^{\Theta }= & \{\{R_{1}\},\{R_{2}\},\{I_{1}\},\{I_{2}\},\\ & \{R_{1},R_{2}\},\{I_{1},I_{2}\},\\ & \{R_{1},I_{1}\},\{R_{1},I_{2}\},\{R_{2},I_{1}\},\{R_{2},I_{2}\},\\ & \{R_{1},R_{2},I_{1}\},\{R_{1},R_{2},I_{2}\},\{R_{1},I_{1},I_{2}\},\{R_{2},I_{1},I_{2}\},\\ & \{R_{1},R_{2},I_{1},I_{2}\}=\Theta ,\\ & \emptyset \}. \end{array}$$

The initial belief function is determined based on the Dirichlet distribution [53], and the initial preference for irregular forms of individuals can be controlled by the parameter φ. The parameters are below unless stated otherwise. An equal initial belief function without any preference φ=1 is applied. The activation probability is f=10%, the reliability function is $r_{max}=0.95$ and $r_{\eta }=0.2$, the self-learning is not considered ($p_{s}=100\%$ and α=0) but forgetting is considered (η=2, $p_{f}=5\%$ and β=4%). The results are the average of 200 independent simulations. The simulations were conducted on a computer equipped with an 11th Gen Intel(R) Core(TM) i7−1165G7 processor at 2.80 GHz and 16 GB of RAM.

### 3.1. Network structure

Three representative synthetic networks are employed to examine the impact of network topology on language dynamics, including Watts–Strogatz (WS) small-world network, Erdos–Rényi (ER) random network and Barabási–Albert (BA) scale-free network [22]. In the WS network, each node is connected to k=10 nearest neighbours, and the probability for rewiring each edge is $p_{r}=0.1$. The ER network has an edge creation probability $p_{c}=0.1$. In the BA network, the number of edges to attach from a new node to existing nodes is $n_{e}=5$. All three networks consist of |N|=100 nodes, resulting in a similar network density of approximately 0.1.

To assess and compare the convergence and consensus process of individuals in the network, two indices are defined. The first index is the ratio of individuals $L_{n}(t)$ who have already achieved convergence at the nth dominant focal element, which typically corresponds to the singleton proposition, at time t. The first/most dominant focal element is most often focused on, which can be obtained by $b_{1}={\arg\;\max}_{\chi \in \Theta }\rho_{\chi }(\infty )$, where $\rho_{\chi }(\infty )$ signifies the ratio of individuals adhering to belief χ upon stabilization. In this case, the ratio of individuals who have converted at the first dominant focal element can be obtained by


(3.1) $$\begin{array}{ccc} & L_{1}(t)=\sum_{i\in N}\delta \left(m_{i}^{\left(t\right)}(b_{1})=1\right)/|N|, & \end{array}$$

where δ is a Boolean function and m(A) measures the degree of belief exactly assigned to the proposition A. $L_{1}(\infty )=1,L_{j}(\infty )=0,\forall j$ implies that all individuals have attained linguistic consensus in the network.

The second index is the belief index (BI) R(t), which is defined by


(3.2) $$R(t)=\frac{\sum_{i\in N}\left(m_{i}^{\left(t\right)}(b_{1})-\sum_{j\neq 1}m_{i}^{\left(t\right)}(b_{j})\right)}{|N|},$$

where b denotes a singleton proposition, and $m_{i}^{\left(t\right)}(b_{j})$ signifies individual i’s belief function regarding $b_{j}$ at time t. It is important to note that R(∞)=1 reflects linguistic consensus within the network while R(∞)=0 indicates that half of the individuals reach the same proposition, as opposed to none.

**Figure 3.**
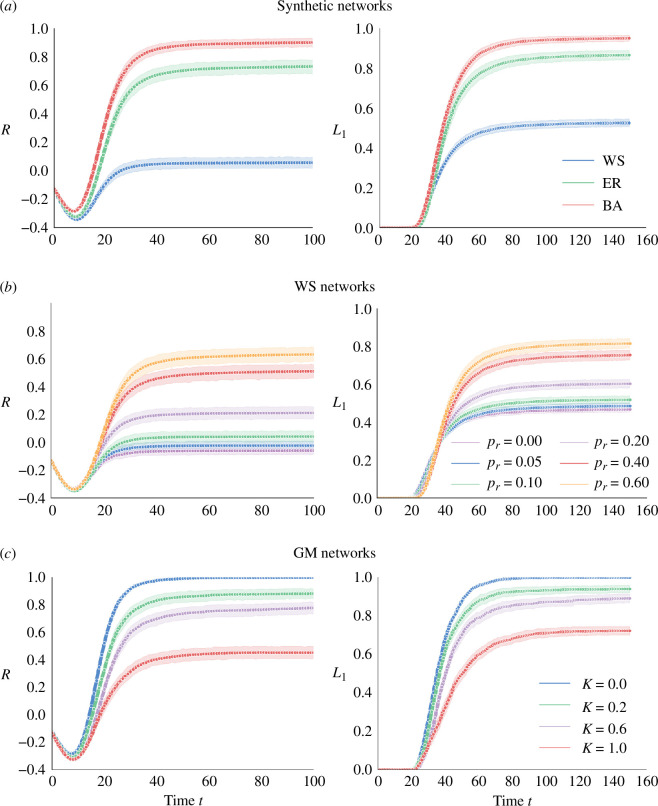
Impact of network structure on belief convergence process, including (*a*) three typical synthetic networks, (*b*) WS networks with varying rewiring probabilities $p_{r}$ and (*c*) a family of GM networks.

The comparative analysis of the three typical synthetic networks is illustrated in figure 3*a*. It is worth noting that R(t)<0 occurs because the belief function of $b_{1}$ may be lower than the sum of the belief function of other propositions at the beginning. In the case of WS networks, $L_{1}(\infty )\approx 0.5$ and R(∞)≈0 signify that only approximately half of the individuals’ beliefs converge towards $b_{1}$. A discerning observation reveals that both indices in WS networks consistently remain significantly lower than those observed in ER and BA networks. This discrepancy suggests that individuals in the WS network, which closely resembles real social networks, tend to adhere to their own beliefs rather than follow the beliefs of the crowd, leading to polarization and fragmentation. In contrast, both indices in the ER and BA networks reach a higher value, indicating individuals can easily reach consensus due to the equally distributed edges in the ER network and a small number of highly influential individuals generated by the preferential attachment mechanism in the BA network. The indices consistently exhibit the highest values in the BA network that are close to 1.

Moreover, we delve into WS networks with varying rewiring probabilities in figure 3*b*. When $p_{r}=0$, the network is a regular lattice characterized by a high average clustering coefficient and long average path lengths. As $p_{r}$ increases, the network gradually adopts small-world characteristics. In this process, the average clustering coefficient decreases slowly, while the average path length decreases rapidly, facilitating more efficient communication and information exchange among individuals. When $p_{r}$ is close to 1, it becomes a random network. In the case of a regular lattice ($p_{r}=0$), achieving consensus becomes challenging, as evidenced by the lowest values of $L_{1}<0.5$ and R<0 among all scenarios. Both $L_{1}$ and R increase with the rise of $p_{r}$, and when $p_{r}=0.6$, both approach values similar to those observed in the ER network. This trend indicates the significant role played by the average clustering coefficient in influencing language dynamics. This observation highlights the pivotal role played by the high average clustering coefficient, possibly coupled with degree assortativity, in preventing individuals from reaching consensus. Consequently, the WS network, characterized by its tendency to ‘split’ individuals into several distinct factions, struggles to facilitate effective communication and discussion. This inherent division makes it challenging to achieve unified beliefs, that is, consensus, within the network.

To provide a deeper understanding of the influence of network structure, we incorporate a one-parameter family of networks with tunable degrees of heterogeneity, as developed by Gómez-Gardeñes & Moreno [41], referred to as the GM network, into this study. This model allows the construction of networks with the same average connectivity ⟨d⟩, ensuring an equal number of nodes and edges. The structural characteristics of the network interpolate smoothly from those of a BA network to an ER network by varying a parameter, κ, which ranges from 0 to 1. Specifically, when κ=1, the network mirrors an ER random network, while κ=0 yields a BA scale-free network. The degree of network heterogeneity escalates as κ decreases from 1 to 0. The results of this investigation are depicted in figure 3*c*. It can be found that the highest values of $L_{1}$ and R manifest in the BA network (κ=0), while the lowest values materialize in the ER network (κ=1), consistent with the findings illustrated in figure 3*a*. This reaffirms that individuals within a BA network can readily achieve consensus. In addition, as network heterogeneity increases, characterized by a declining κ value, individuals are more likely to converge towards a shared belief, ultimately reaching a consensus. This observation demonstrates the role of network heterogeneity in promoting consensus among individuals, a phenomenon with substantial societal benefit [54]. In conclusion, the network topology plays a significant role in shaping language dynamics. The structure and characteristics of networks can impact the extent to which individuals reach consensus and converge on linguistic rules. These findings offer valuable insights into the dynamics of language and communication within complex social networks.

**Figure 4.**
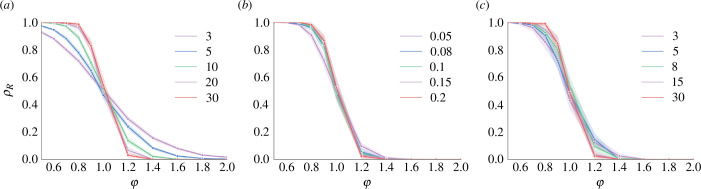
Impact of initial belief function controlled by φ and network density controlled by (*a*) k={3,5,10,20,30} in the WS network, (*b*) $p_{c}=\{0.05,0.08,0.1,0.15,0.2\}$ in the ER network and (*c*) $n_{e}=\{3,5,8,15,30\}$ in the BA network.


Furthermore, the impact of the network density and the initial belief function on the regularization process is depicted in figure 4, where

(3.3) $$\rho_{R}=\sum_{i=1}^{n}\rho_{R_{i}}(\infty ),$$

where $\rho_{R}$ indicates the proportion of individuals adopting regular forms after stabilization. It can be found that, when a larger proportion of individuals initially prefer regular forms (indicated by a smaller value of φ), more individuals will eventually converge to prefer regular forms, regardless of the network density. The density does not have a significant impact on the regularization process. However, it is noteworthy that the density of the WS network, as shown in figure 4*a*, has a more pronounced impact compared to that of the ER and BA networks.

### 3.2. Community structure

As discussed earlier, information exchange and consensus-reaching within the network can be impeded when the network is fragmented into distinct factions. It has been found that an interconnectivity parameter can cause consensus or polarization among different communities in the majority model [55]. In this section, we further explore the influence of community structure. In addition to synthetic networks, we incorporate two real networks known for their distinct community structures, alongside the utilization of the LFR benchmark networks, shedding light on the role of community structure in shaping language dynamics. In detail, Zachary’s karate club represents the friendship between 34 members of a karate club, who are split into two parts because of a conflict between the administrator and instructor. American College football network describes American football games between Division IA colleges that belong to 12 conferences during regular season Fall 2000. Additionally, the widely used LFR benchmark network with a clear community structure [42] is included in this study. The networks consist of |N|=100 nodes. The power law exponent for the degree distribution and the community size distribution are $\tau_{1}=3$ and $\tau_{2}=1.1$, respectively. The fraction of inter-community edges incident to each node is μ=0.1. The average degree and maximum degree are 10 and 50, respectively. The minimum and maximum sizes of the community are 30 and 50, respectively. In addition, we only analyse the condition that there are three communities. To assess the degree of consensus within the community, we employ the local belief index r(t),


(3.4) $$r(t)=\frac{\sum_{c_{k}\in C}\frac{\sum_{i\in c_{k}}\left(m_{i}^{\left(t\right)}(b_{1}^{\left(k\right)})-\sum_{j\neq 1}m_{i}^{\left(t\right)}(b_{j}^{\left(k\right)})\right)}{|c_{k}|}}{|C|},$$

where C indicates the set of community, $c_{k}$ represents the kth community, and $b_{1}^{\left(k\right)}$ is the most dominant focal element in community $c_{k}$.

**Figure 5.**
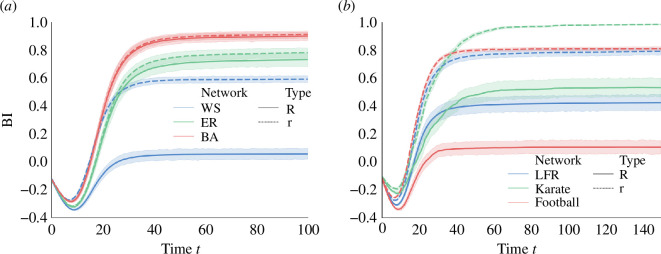
Impact of community structure on the belief index, illustrated in (*a*) synthetic networks and (*b*) networks with known community structure.

Illustrated in figure 5, an intriguing observation emerges: r consistently surpasses R for all cases when the system stabilizes. This is particularly pronounced when R attains smaller values. For instance, in the WS network, despite R≈0 in the stabilized state, r is around 0.6. In the LFR network, with R≈0.4, r achieves a value of approximately 0.8. Remarkably, even in the two real-world networks, a substantial consensus prevails. In the football network, r reaches approximately 0.8 when R≈0, and in the karate network, r attains a near-unanimous consensus and hovers at r≈1 when R≈0.6. In contrast, in the ER and BA networks, where all members already exhibit a notably high belief index R, the disparity between R and r remains relatively small, even when r surpasses R. Collectively, these findings highlight the pivotal role of community structure in preventing linguistic consensus among individuals within the network. The presence of well-defined communities within networks can respectively promote and hinder consensus among individuals within and outside the community. In social dynamics, when stylized opinions evolve over time, community structures emerge, indicated by clusters of consensus-reaching individuals, which was also reflected in naming game models [56].

### 3.3. Self-learning and forgetting

**Figure 6.**
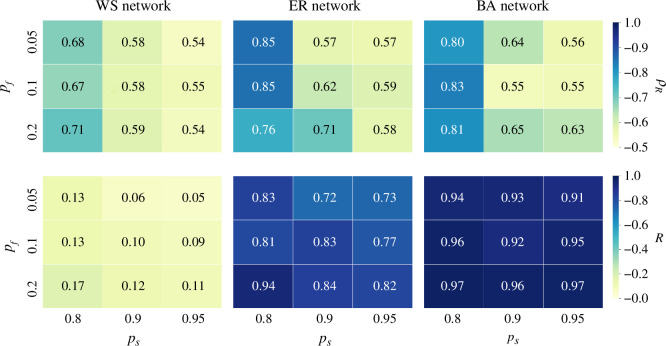
Impact of self-learning $p_{s}$ and forgetting $p_{f}$ behaviours on the regularization (measured by $\rho_{R}$ in the upper row) and consensus process (measured by the belief index R(∞) in the lower row), illustrated in three synthetic networks.

The effects of self-learning and forgetting behaviours are further explored in different types of synthetic networks to gain valuable insights into the dynamics of linguistic regularization and consensus. The learning rate is α=5%, while the probabilities of social learning $p_{s}$ and forgetting $p_{f}$ vary across a specified range, as depicted in figure 6. It can be found that self-learning, represented by decreasing values of $p_{s}$, can greatly enhance the regularization process, that is, more individuals adopt the regular forms after stabilization, quantified by $\rho_{R}$. The biased child learners, who prefer regular forms, reinforce their beliefs on regular forms, thereby leading to a stronger regularization process. On the contrary, the impact of forgetting, represented by increasing values of $p_{f}$, is less pronounced and does not follow a consistent pattern. Specifically, forgetting reduces the regularization process in ER networks when $p_{s}=80\%$ but enhances regularization in ER networks when $p_{s}=90\%$ and WS networks when $p_{s}=80\%$, while its impact is less evident in other scenarios. Regarding the consensus process, both forgetting and self-learning behaviours enhance consensus, as measured by the belief index R(∞). However, this enhancement is not as strong as in the regularization process.

In general, both the extent of regularization and the consensus process are stronger in ER and BA networks compared with WS networks. In particular, the extent of consensus is notably stronger in BA networks, with R(∞)≈1 in all cases. This difference in language dynamics can be attributed to the distinctive structural characteristics of networks, as previously discussed. Overall, the findings presented in this section shed light on the interplay between self-learning, forgetting and network structure in shaping language dynamics.

## 4. Discussion

The acquisition of linguistic rules and irregular exceptions among individuals has been a long-standing topic of debate in cognitive science. Over the years, numerous agent-based models have been introduced to capture the dynamics of repeated communication and learning behaviours within society. In this study, we employ a generalized Bayesian inference approach to shed light on the intricate social learning processes of individuals and extend the naming game model to incorporate the topological structure of social networks. We introduce significant social behaviours, including self-learning with preferences and forgetting under memory constraints, providing a more realistic representation of individual behaviour. Additionally, we emphasize the importance of assessing an individual’s role based on both intrinsic factors and external factors, as these factors affect the degree to which individuals influence each other.

Our investigation spans synthetic networks and real-world social networks to demonstrate the profound impact of various topological structures. These insights offer valuable perspectives on the influence of network topology on language dynamics. We observed that WS small-world networks, which tend to ‘split’ individuals into distinct factions, often lead to polarization and fragmentation of beliefs rather than consensus. This finding suggests that real-world networks may encounter similar challenges in achieving unified beliefs. In contrast, in BA scale-free networks, driven by the preferential attachment mechanism, and ER random networks, where edge existence is governed by independent probabilities, people are more likely to reach consensus, with a particularly high consensus degree in the BA network, demonstrating the impact of the clustering coefficient. The introduction of the GM network with the same average connectivity and tunable heterogeneity highlights the facilitating role of network heterogeneity in fostering consensus, with potential societal implications, especially in the context of opinion dynamics and decision-making. Therefore, our study provides invaluable insights into understanding and predicting collective language behaviour when social networks exhibit different structural characteristics.

In addition, we conducted experiments in real-world social networks with known community structures and LFR benchmark networks, revealing that consensus among individuals within a community is significantly higher than that among individuals in the entire network. This highlights the critical role of community structure in language dynamics, indicating that individuals are more likely to align with the prevailing opinions of insiders in tightly connected groups. Overall, this work demonstrates the intricate interplay between network structure and language dynamics, offering beneficial insights for group leaders, organizers and policymakers aiming to foster consensus and effective communication and decision-making within groups. For example, in order to improve communication and consensus-reaching in various social contexts, the pairwise interaction between certain individuals can be rewired to adjust network heterogeneity and community structure, which actually reflects the importance of the network structure composed of individuals.

Furthermore, we found that self-learning with preferences can significantly enhance the regularization process within the network, while forgetting has a relatively minor impact. These insights advance our understanding of how language regularization and consensus evolve within different social and communication contexts, where individuals may exhibit varying degrees of memory and self-learning capabilities. This study will provide insights into how individual learners adopt language rules through the lenses of complexity science and decision science, offering a fresh perspective on the language dynamics in society.

## Data Availability

All networks (or datasets) used in this work are created by Python script, and the way to create them is illustrated in the main text. The code can be found in Zenodo via this link [57].

## Basic concepts of Dempster–Shafer theory

The Dempster–Shafer theory, developed and formalized by Dempster and Shafer in the 1960s [44,45], provides a powerful tool for reasoning with uncertainty. It extends traditional probability theory by allowing beliefs to be assigned to sets of events rather than just individual events.

Let $\Theta =\{\theta_{1},\theta_{2},\cdots \;,\theta_{N}\}$ represent a finite set of N mutually exclusive and collectively exhaustive elements, i.e. that account together for all possible outcomes. In the frame of discernment Θ, the elements satisfy the condition $\theta_{i}\cap \theta_{j}=\emptyset ,\forall i\neq j,i,j\in \{1,2,\cdots \;,N\}$, where ∅ indicates the empty set. This set describes all possible states or all possible hypotheses being considered. For example, when detecting the colour of a traffic light, the frame of discernment can be defined as Θ={Green,Yellow,Red}, encompassing the three colours (possible states): green, red and yellow. However, when the traffic light malfunctions, multiple colours may be illuminated simultaneously. In such cases, the power set of the frame of discernment, denoted as $2^{\Theta }$ or P(Θ), is used to account for all possible combinations of hypotheses, including the empty set ∅ and Θ itself:

$$2^{\Theta }=P(\Theta )=\{\emptyset ,\{\text{Green}\},\{\text{Yellow}\},\{\text{Red}\},\{\text{Green},\text{Yellow}\},\{\text{Green},\text{Red}\},\{\text{Yellow},\text{Red}\},\{\text{Green},\text{Yellow},\text{Red}\}\}.$$

In general, the power set of Θ is defined as follows:

$$\begin{array}{c} P(\Theta )=2^{\Theta }=\{\emptyset ,\{\theta_{1}\},\{\theta_{2}\},\cdots \;,\{\theta_{N}\},\{\theta_{1},\theta_{2}\},\cdots \;,\{\theta_{1},\theta_{N}\},\cdots \;,\{\theta_{2},\theta_{3},\cdots \;,\theta_{N}\},\Theta \}, \end{array}$$

which includes all subsets of the frame of discernment Θ. Subsets of Θ that contain only one element are known as singletons.

In Dempster–Shafer theory, beliefs are assigned to subsets of hypotheses in the power set rather than individual hypotheses, allowing for the representation of both specific and ambiguous information. The basic probability assignment, also known as the belief function or mass function, is a mapping function $m:2^{\Theta }\to [0,1]$, satisfying

$$m(\emptyset )=0,\sum_{A\subseteq \Theta }m(A)=1.$$

The belief function m(A) quantifies the level of support assigned exactly to the subset A and cannot be assigned to any smaller subset. Typically, the sum of the belief functions assigned to the subsets of Θ is unity, indicating that no belief is left to the empty set m(∅)=0. The belief function assigned exactly to Θ indicates the degree of global ignorance. If m(Θ)=1 (i.e., m({Green,Yellow,Red})=1 in the example of the traffic light), it implies that no specific information and knowledge is available, because no belief is assigned to any proper subsets. The belief function assigned to the subsets of θ other than Θ or any singletons indicates the degree of local ignorance.

When there is no local nor global ignorance, the belief function reduces to the classic probability function. Overall, the power set in Dempster–Shafer theory allows for a more reasonable representation of uncertainty by enabling evidence to be distributed over all possible combinations of hypotheses. This represents a more general framework compared with traditional probability theory, which directly assigns probabilities to individual hypotheses. Hence, the power set can model both ignorance and specific knowledge, making Dempster–Shafer theory a powerful tool for reasoning under uncertainty. Further details on Dempster–Shafer theory, such as the combination rule, can be found in [44,45,50], because they are not directly relevant to this study.
